# Using Bayesian methodology to explore the profile of mental health and well-being in 646 mothers of children with 13 rare genetic syndromes in relation to mothers of children with autism

**DOI:** 10.1186/s13023-018-0924-1

**Published:** 2018-10-25

**Authors:** Dawn Adams, Richard P Hastings, Clair Alston-Knox, Rina Cianfaglione, Kate Eden, David Felce, Gemma Griffith, Jo Moss, Chris Stinton, Chris Oliver

**Affiliations:** 1 Cerebra Centre for Neurodevelopmental Disorders, School of Psychology, University of Birmingham, Birmingham, B15 2TT UK; 2 Autism Centre of Excellence, School of Education and Professional Studies, Griffith University, Brisbane, Australia; 3 Centre for Educational Development Appraisal and Research, University of Warwick, Warwick, UK; 4 Griffith Social and Behavioural Research College, Griffith University, Brisbane, Australia; 5 Welsh Centre for Learning Disabilities, Cardiff University, Cardiff, UK; 6 School of Psychology, Bangor University, Bangor, UK

**Keywords:** Syndrome, Mothers, Mental health, Positive mental health, Genetic syndrome

## Abstract

**Background:**

It is well documented that mothers of children with intellectual disabilities or autism experience elevated stress, with mental health compromised. However, comparatively little is known about mothers of children with rare genetic syndromes. This study describes mental health and well-being in mothers of children with 13 rare genetic syndromes and contrasts the results with mothers of children with autism.

**Methods:**

Mothers of children with 13 genetic syndromes (*n* = 646; Angelman, Cornelia de Lange, Down, Fragile-X, Phelan McDermid, Prader-Willi, Rett, Rubenstein Taybi, Smith Magenis, Soto, Tuberous Sclerosis Complex, 1p36 deletion and 8p23 deletion syndromes) and mothers of children with autism (*n* = 66) completed measures of positive mental health, stress and depression. Using Bayesian methodology, the influence of syndrome, child ability, and mother and child age were explored in relation to each outcome. Bayesian Model Averaging was used to explore maternal depression, positive gain and positive affect, and maternal stress was tested using an ordinal probit regression model.

**Results:**

Different child and mother factors influenced different aspects of mental well-being, and critically, the importance of these factors differed between syndromes. Maternal depression was influenced by child ability in only four syndromes, with the other syndromes reporting elevated or lower levels of maternal depression regardless of child factors. Maternal stress showed a more complex pattern of interaction with child ability, and for some groups, child age. Within positive mental health, mother and child age were more influential than child ability. Some syndromes reported comparable levels of depression (SMS, 1p36, CdLS) and stress (SMS, AS) to mothers of children with autism.

**Conclusions:**

Bayesian methodology was used in a novel manner to explore factors that explain variability in mental health amongst mothers of children with rare genetic disorders. Significant proportions of mothers of children with specific genetic syndromes experienced levels of depression and stress similar to those reported by mothers of children with autism. Identifying such high-risk mothers allows for potential early intervention and the implementation of support structures.

**Electronic supplementary material:**

The online version of this article (10.1186/s13023-018-0924-1) contains supplementary material, which is available to authorized users.

## Background

Mothers of children with neurodevelopmental disorders and/or intellectual and developmental disabilities (IDD) report elevated levels of stress and affective symptoms when compared to mothers of typically developing children [1–3]. These differences in maternal well-being are established by the time children are 3–5 years of age [3, 4] and continue over time [5].

Given these elevated levels of stress and affective symptoms, an important question is whether there are particularly high-risk groups of mothers that might be targeted for early support and hence whether the nature of a child’s neurodevelopmental disability is associated with compromised mental health. To date, the focus has been on mothers of children with autism, with consistent reports of higher levels of mental health difficulties and stress when compared to mothers of children with other neurodevelopmental disorders, including general IDD [3, 4, 6]. A meta-analysis of such studies confirms that whilst mothers of children with autism are more impacted by parenting stress, the extent to which they differ from other mothers varies considerably depending upon the comparison groups [7]. The majority of research has focused upon mothers as they tend to be the primary caregivers. The limited studies that have included or focused upon fathers draw mixed conclusions with regards to levels of stress compared to mothers and compared to fathers of typically developing children [8].

In contrast to the extensive literature focusing upon the mental health of mothers of children with autism, research into the mental health of mothers of children with known genetic aetiologies associated with neurodevelopmental disorders is comparatively sparse. Although each genetic syndrome affects only a small proportion of people, collectively the prevalence is relatively high [9]. Given that a molecular diagnosis can now be identified for most individuals with severe IDD and that the number of syndromes associated with IDD is increasing with increased genetic techniques (see [10] for a review), further research is needed to understand the pattern of aetiological group differences and factors that may be contributing to this.

Studies investigating maternal mental health in syndromes have focused predominantly on the most common genetic causes of IDD such as Fragile-X syndrome (FXS) and Down syndrome (DS), with relatively consistent results. It was concluded that mothers of children with FXS displayed fewer signs of compromised psychological well-being than mothers of children with autism, but more than mothers of children with DS [11]. Mothers of children with DS report lower levels of stress and negative impact than mothers of children with autism [12] and, in some studies, report levels of mental health problems comparable to those experienced by parents of typically developing children [13]. This raises the possibility that the so-called “Down syndrome advantage” [14] might drive the results of the few studies reporting parental stress in rarer genetic syndromes, as they typically use DS as a contrast group. The findings that parents of children with Williams or Smith-Magenis syndromes report more family problems and pessimism about their child’s future than parents of children with DS [15], and that parents of children with Cornelia de Lange syndrome (CdLS) report higher levels of stress than parents of children with DS [16], must therefore be interpreted in relation to the use of DS as a contrast group. An alternative approach to comparing to DS by comparing levels of parental mental health in three rare genetic syndromes (CdLS, Cri du Chat, and Angelman syndromes [AS]) with parents of children with autism has been suggested [17], citing them as a useful benchmark high-stress comparison group. This strategy is adopted in this study.

The model of “direct” and “indirect” effects of specific syndromes and behaviour [18] can be used to hypothesise why syndrome-associated differences in maternal mental health may result. A genetic syndrome may predispose an individual to display certain behaviours or patterns of behaviour; described as the “direct” effect of the syndrome. These behaviours may evoke particular reactions from others, which is described as the “indirect” effect of the syndrome (discussed further in [19]). Syndrome characteristics may influence child presentation (the “direct” effect) which will then have differing impact upon parenting behaviours and consequences for parental well-being (the “indirect effect”). The available research in DS and FXS supports the possibility that syndromes potentially have an indirect effect on parents. However, the comparative risk for maternal mental health problems in syndromes other than these is difficult to comprehend because studies have tended to use different measures and designs and compare only one or two syndrome groups. Consequently, this limits the extent to which the available studies on the rarer syndromes can be compared with each other.

The main aim of the present study is to address the methodological limitations of existing research into the mental health of parents of children with genetic syndromes associated with neurodevelopmental disorders or disabilities. To do so, we recruited families of children with 13 different genetic syndromes and used the same measures with each group. In order to benchmark the levels of maternal well-being in these 13 syndromes and address the question of whether any of the syndrome groups in this study represent an especially high-risk group, the results were compared with those of a group of mothers of children with autism. Autism was chosen as the high-risk comparison group as multiple population-based studies as well as systematic reviews and meta-analyses [3, 7, 20–23] have identified parents of children with autism as being at higher risk of mental health difficulties than parents of children without autism, children with rare genetic syndromes, children with other disabilities and typically developing children. Effect sizes for this increased risk are consistently large, for example, [7]‘s meta-analysis of studies focusing upon parent stress in parents of children with autism report an effect size of 1.54 for comparisons of parents of children with autism to parents of typically developing children and 0.64 for comparisons to parents of children with other disabilities. The combination of the high volume of research from across the world with the consistent finding of elevated levels of maternal mental health difficulties provides a strong rationale for using autism as a “high-risk” comparison group against which the other syndromes can be compared.

In addition to a focus on mental health problems, we are mindful of the growing research base regarding the positive impact of caring for an individual with IDD [24, 25]. Intriguingly, a population-based study [3] found no disability group differences and no evidence of differences compared to mothers of children without disabilities for maternal positive mental health. A similar pattern of no group differences for positive impact has been found in genetic syndrome family research studies [12, 26], but the research remains limited.

Therefore, we included both negative and positive well-being measures in the present research to answer the following research questions:

1. What are the profiles of positive mental health, stress and depression in mothers of children with a range of rare genetic syndromes and how do these compare with mothers of children with autism?
2. Does genetic syndrome predict levels of maternal mental health and if so, is this more predictive than basic child variables that differ between the syndromes, such as level of ability?

## Methods

### Participants

For all of the syndrome groups included in the study with the exception of Rett syndrome (RTT) (AS, CdLS, DS, FXS, Phelan-McDermid [PMS], Prader-Willi [PWS], Rubinstein-Taybi [RTS], Soto, Smith-Magenis [SMS], Tuberous Sclerosis Complex [TSC], 1p36 deletion syndrome, 8p23 deletion syndrome and the autism group [ASD]), questionnaire packs were distributed to members of each syndrome support group within the UK. [27] provides detailed information on the recruitment process but briefly, questionnaire packs were provided to the syndrome support groups who then sent them out to their members. The research team only received information on demographics, child and family variables from the participants who consented and returned their questionnaire. The 87 mothers of daughters with RTT were recruited in a study which used the British Isles Rett Syndrome Survey (BIRSS) as a sampling frame (see [28] for further details).

Caregivers from 816 families responded and consented to take part. Due to small numbers and the lack of ability to examine differences across groups, thirty-nine (3.3%) were excluded because they were not the child’s mother or adoptive mother (i.e. were fathers, grandparents, foster carers or paid carers) and 11 (1.2%) were excluded as the child was aged under 2 years. Respondents who completed fewer than 75% of items in the questionnaires of interest to this study were excluded to minimise the impact of missing data. As per [27], data were excluded if the participant did not have a genetic diagnosis from a clinical geneticist, pediatrician, neurologist or psychiatrist. In relation to the ASD group, participants who had scores below the cut-off of 15 on the Social Communication Questionnaire (SCQ) [29] were excluded to provide additional confidence in the reported diagnoses. To reduce the risk of potential overlap between the ASD and the syndrome groups, participants in the autism group were asked if their child had any additional diagnoses or a diagnosis of a genetic syndrome. No participants listed any genetic syndrome in addition to the autism diagnosis.

Following exclusions, a sample of 712 mothers was retained for analysis. The sample consisted of 646 mothers of children with one of 13 genetic syndromes and 66 mothers of children with ASD. Table 1 summarises the number, age and gender distribution of the children and age of the mothers. The range in sample sizes partly reflects the range in prevalence rates for the syndromes. For example, the prevalence of AS (1:10,000-40,000) [30] is considerably lower than that for FXS (1:5,000) [31]. The current article does not allow for a description of the genetic cause or phenotype of each individual syndrome but excellent descriptions are available in Table 1 of [32] and within the wider literature.

**Table 1:** Demographic characteristics (age, gender) and descriptive data by syndrome group

	AS	ASD	CdLS	DS	FXS^a^	PMS	PWS	RTT^a^	RTS	SMS	Soto	TSC	1p36	8p23	Total
n	28	66	44	29	102	31	101	87	47	20	38	71	26	22	712
Child mean age (*sd*, range)	10.9 *3.3* (3–15)	15.5 *6.5* (6–42)	12.8 *8.2* (2–45)	25.4 *11.8* (8–45)	15.0 *8.2* (2–42)	11.3 *8.4* (2–37)	12.2 *8.1* (2–45)	20.1 *10.2* (4–47)	21.3 *10.5* (6–53)	11.6 *7.2* (3–32)	15.3 *9.3* (2–43)	18.8 *10.7* (2–50)	10.9 *8.9* (2–39)	10.8 *5.5* (4–21)	15.2 *9.5* (2–53)
% Male child	46.4	86.4	47.7	53.3	100	45.2	52.3	0	55.3	60	68.4	60.6	78.6	68.2	57.0
Maternal mean age *(sd)*	40.8 *4.9*	47.9 *6.8*	45.1 *9.1*	59.1 *12.3*	45.6 *9.5*	42.3 9.9	44.5 *8.5*	50.7 *9.2*	49.8 *9.9*	43.7 *8.5*	46.3 *8.3*	48.1 *10.3*	41.2 *10.8*	39.6 *6.1*	46.6 *9.8*
Speech % verbal	32.1	92.4	68.2	100	94.2	38.7	98	–	83	80	92.1	76.1	57.7	91.9	82.3
Hearing % normal	92.9	98.5	56.8	58.6	97.1	90.1	99.0	–	80.9	55	76.3	94.4	61.5	90.9	87.3
Vision % normal	85.7	86.4	63.6	43.3	88.3	71.0	73.3	–	51.1	75.0	73.7	88.7	26.9	45.5	74.4
Mobility % mobile	39.3	92.4	54.5	100	86.4	71	78.2	–	74.5	70	81.6	78.9	46.2	59.1	76.6
Self-help % able/partly able	14.3	89.2	40.9	90	88.2	22.6	78.2	–	80.9	60	84.2	66.2	42.3	59.1	70.4

^a^Gender distribution can be explained in these groups as only males with FXS were recruited and RTT almost exclusively affects females

*AS* Angelman Syndrome, *ASD* Autism Spectrum Disorder, *CdLS* Cornelia de Lange syndrome, *DS* Down Syndrome, *FXS* Fragile-X Syndrome, *PMS* Phelan McDermid Syndrome, *PWS* Prader-Willi syndrome, *RTT* Rett Syndrome, *RTS* Rubenstein Taybi syndrome, *SMS* Smith Magenis Syndrome, *TSC* Tuberous Sclerosis Complex, *1p36* 1p36 deletion syndrome, *8p23* 8p23 deletion syndrome

### Demographic characteristics

The mean age of the 712 children was 15.2 years (sd 9.5) and the mean age of the mothers was 46.6 years (sd 9.8). Fifty-seven percent of the children were male and 96.9% were the participants’ natural mothers, with the remainder being long-term adoptive mothers. Gender differences were as expected given that only males with FXS were recruited; RTT almost exclusively affects females, and ASD spectrum disorder is more prevalent in males. However, a higher proportion of males than expected was also found in the 1p36 syndrome group. Data from the Wessex questionnaire (see Measures section below) reflect the different physical and cognitive profiles associated with the different syndromes.

### Ethical considerations

Ethical approval was obtained for this study in 2010 from Coventry Research Ethics Committee, as part of a larger ongoing study entitled ‘Understanding behaviour and family adjustment in individuals with neurodevelopmental disorders’ (REC reference number: 10/H1210/01).

All procedures performed were in accordance with the ethical standards of the institutional research committees and with the 1964 Helsinki declaration and its later amendments or comparable ethical standards.

Informed consent was obtained from individuals who had the capacity to provide consent themselves. For those who did not have capacity to provide their own consent, parents or carers were able to act as consultees. Consultees were asked to advise on what the wishes of the individual would be if they were able to consent from themselves.

### Measures

A background questionnaire was used to collect demographic information and, for the ASD group, included the Social Communication Questionnaire [29]. The Wessex Scale [33] was also used to collect data on each child’s mobility, self-help scores, speech, vision and hearing. Within this study, the Wessex self-help scale was used as a proxy measure of ability (as per [34, 35]). The mothers then completed the following four questionnaires addressing dimensions of their well-being.

The Positive Gain Scale (PGS) [36] comprises seven items to assess the direct positive aspects of having a child with a disability, such as “since having this child I feel I have grown as a person”. Each item is rated on a 5-point Likert scale. These scales were reverse coded for this study, so the higher the score, the more positive gains reported by the participant. Internal consistency is good, with Cronbach’s Alpha being reported as .71 [17] and .93 within the current sample.

To minimise demands upon mothers, the Positive Affect Scale-5 (PAS5) was used (as per [17]). This comprises five items from the Positive Affect Scale (PANAS) [37] with the highest total item correlations. Correlation of the full 10-item Positive Affect Scale and the Positive Affect Scale-5 as moderate to strong (*r* = .60) has been reported [17]. Participants were presented with five descriptive words, such as “strong” and “interested” and asked to rate the extent to which they felt this way over the past week on a Likert-type scale. Cronbach’s Alpha was .86 within the current sample.

The seven items assessing depression were selected from the Hospital Anxiety and Depression Scale (HADS) [38]. Although this measure was published in 1983, it continues to have good test-retest reliability and concurrent validity data reported at subscale and total score levels for parents of children with neurodevelopmental disorders [39, 40]. In the present sample, Cronbach’s Alpha was strong (.89).

The Parent and Family Problems Subscale from the Questionnaire on Resources and Stress – Short Form (QRSF) [41], was used to measure general stress associated with raising a child with intellectual disabilities. The five items assessing depression were excluded to reduce potential overlap (as per [17]). This measure continues to be used and reported in recent studies of children with a range of disabilities (e.g. [42, 43]). Mothers were asked to circle “true” or “false” on seven items, such as “other members of the family have to do without things because of <name>”. The Kuder-Richardson coefficient for this version of the questionnaire was .82 within the current sample.

### Statistical analysis

Bayesian methods are emerging in developmental research, and in psychological research more broadly [44]. The approach has been described and discussed in articles in a range of journals relevant to the field of intellectual disability research (e.g. [44–46]). In brief, Bayes’s theorem is a model for learning from data, and consequently, the Bayesian paradigm interprets probability as the subjective experience of uncertainty [47]. As noted [46], Bayesian methods are not meant to test whether a null hypothesis should be rejected, but aim to capture the strength of the evidence for specific hypothesised beliefs. Instead of assuming that there is a fixed but unknown parameter of interest (e.g. one true mean, one true regression coefficient), the Bayesian view is that all unknown parameters should be treated as uncertain, and therefore should be described by a probability distribution [44].

Bayesian analysis formulates this probability distribution for the parameters (and functions of the parameters) using a combination of data (a likelihood distribution) and a prior distribution, which represents the researchers’ knowledge prior to data collection. Although there is a wealth of literature documenting elevated stress and depression in mothers of children with ASD, there is comparatively little on mothers of children with genetic syndromes, particularly relating to positive mental health. Given this, it was decided that there was insufficient information across all the syndromes in this study to set an informative prior belief for how each syndrome may differ, so the hypothesised belief was that there is no difference on measures of mental health between the syndrome groups and ASD. The prior distribution for parameters for syndromes other than ASD was set as a normal distribution centered around zero, and the variance was set to allow variation from ASD. The ASD group was used as the base-level parameter, and a comparison and marker for elevated difficulties was modelled.

The data analysis was undertaken in three stages for each measure of maternal mental health except stress, where only the first two stages were undertaken due to the data being measured on a dichotomous scale (for which Bayesian model averages have not yet been developed). The continuous measure scale data were first analysed as a linear regression where only syndrome was considered, using a Bayesian framework. The linear regression model used in stage 1 is represented by:

$$ {\displaystyle \begin{array}{l} Measure\sim N\left( X\beta, {\sigma}^2\right)\\ {} X\beta ={\beta}_{Autism}+{\beta}_{syndrome\_ difference\_ autism}\\ {}p\left({\beta}_{Austism}\right)\sim N\left({\eta}_A,{\varepsilon}_A^2\right)\\ {}p\left({\beta}_{Syndrome\_ difference\_ autism}\right)\sim N\left({\eta}_B,{\varepsilon}_B^2\right)\\ {}p\left({\sigma}^2\right)\sim InvGamma\left(v,\omega \right)\end{array}} $$

In this framework, the likelihood of the measure of interest (depression, positive gain and positive affect) is considered to be normally distributed, with a mean based on the regression model parameters, the explanatory data and constant variance. The unknown regression parameters (β) have a prior distribution that is also normal, with hyperparameters for the mean and variance given in Table 2. The error variance, σ^2^ is modelled using an inverse gamma prior distribution.

**Table 2:** Fixed hyperparameters for the prior distributions of linear regression coefficients β_ASD_ and β_Syndrome_difference_ASD_ and the variance (σ^2^) for the continuous measures of maternal mental health

	Depression	Positive Gain	Positive Affect
η_A_	7.6	21.0	15.0
ε_A_	0.5	2.0	0.5
η_B_	0.0	0.0	0.0
ε_B_	2.0	2.0	2.0
ν	0.001	0.001	0.001
ω	0.001	0.001	0.001

Computation was performed using Markov Chain Monte Carlo (MCMC) estimation with the MCMCregress function from the MCMCpack library [48] in R [49]. The results were then presented graphically and grouped using the estimated posterior distributions that were statistically similar. Within each figure, shadings were used to represent groupings with similar posterior estimates and credible intervals of 80%, 50% and 20% provided.

As a Bayesian regression model determined that Wessex score, child and maternal age varied between groups, these factors were entered into a classification and regression tree (CART) to explore their impact, if any, on the measure of maternal well-being. This CART analysis informed the final stage of the analysis by highlighting potential interactions and allowing us to estimate suitable factorisation levels, such as carer age, on continuous data. Continuous data are unlikely to change gradually as a regression model would imply, but are more likely to cluster into a category. A Bayesian model averaging (BMA) approach was used to assess the relative influence of each of these factors on maternal mental health. BMA is an extension of the Bayesian inference methods that consider both model and parameter uncertainty. Instead of selecting one particular model as “true”, BMA combines weighted fitted values from multiple models to estimate the posterior distribution of the model parameters. Using this technique, a posterior mean (the value expected under the BMA), posterior standard deviation and the probability of a variable’s inclusion are provided. The probability of inclusion reflects how certain the model is that the coefficient is not zero, taking into account model uncertainty and the other variables. An inclusion probability of 1 indicates 100% certainty that the factor should be included in the model, and an inclusion probability of .5 indicates 50% certainty [50]. BMA computation was estimated using the R BAS library [51] with the default prior distributions for all parameters.

Maternal stress was measured on a scale derived from dichotomous variables, so an ordinal probit regression model was fitted using an MCMC scheme. Vague priors were employed to model the transition of individuals from one stage of stress to another in an ordered sequence. Estimation was conducted using the MCMCoprobit function from the MCMCpack library [48] in R [52], using the algorithm proposed by [53].

## Results

**Fig. 1:**
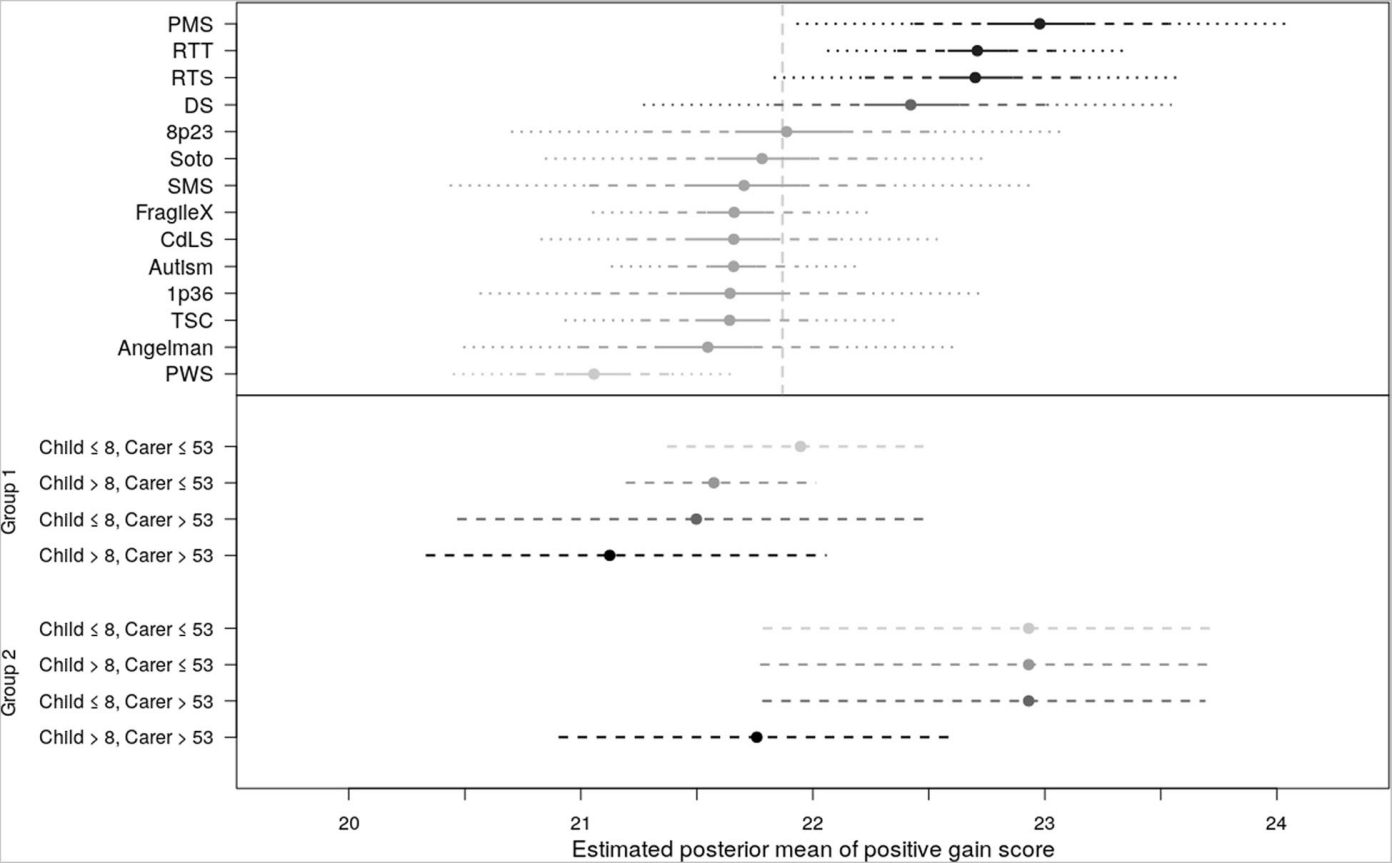
Positive Gain by syndrome group (upper panel) and by BMA groupings (lower panel). Upper panel: Estimated posterior mean of Positive Gain by syndrome group. Extended lines represent Credible Intervals: solid 20%, dashed 50%, dotted 80%. Vertical dashed line is mean of all participants. Lower panel: Posterior probabilities of Positive Gain based on BMA. Group 1: Autism, AS, CdLS, FXS, PWS, SMS, Soto, 1p36, 8p23, TSC. Group 2: DS, RTS, PMS, RTT

**Fig. 2:**
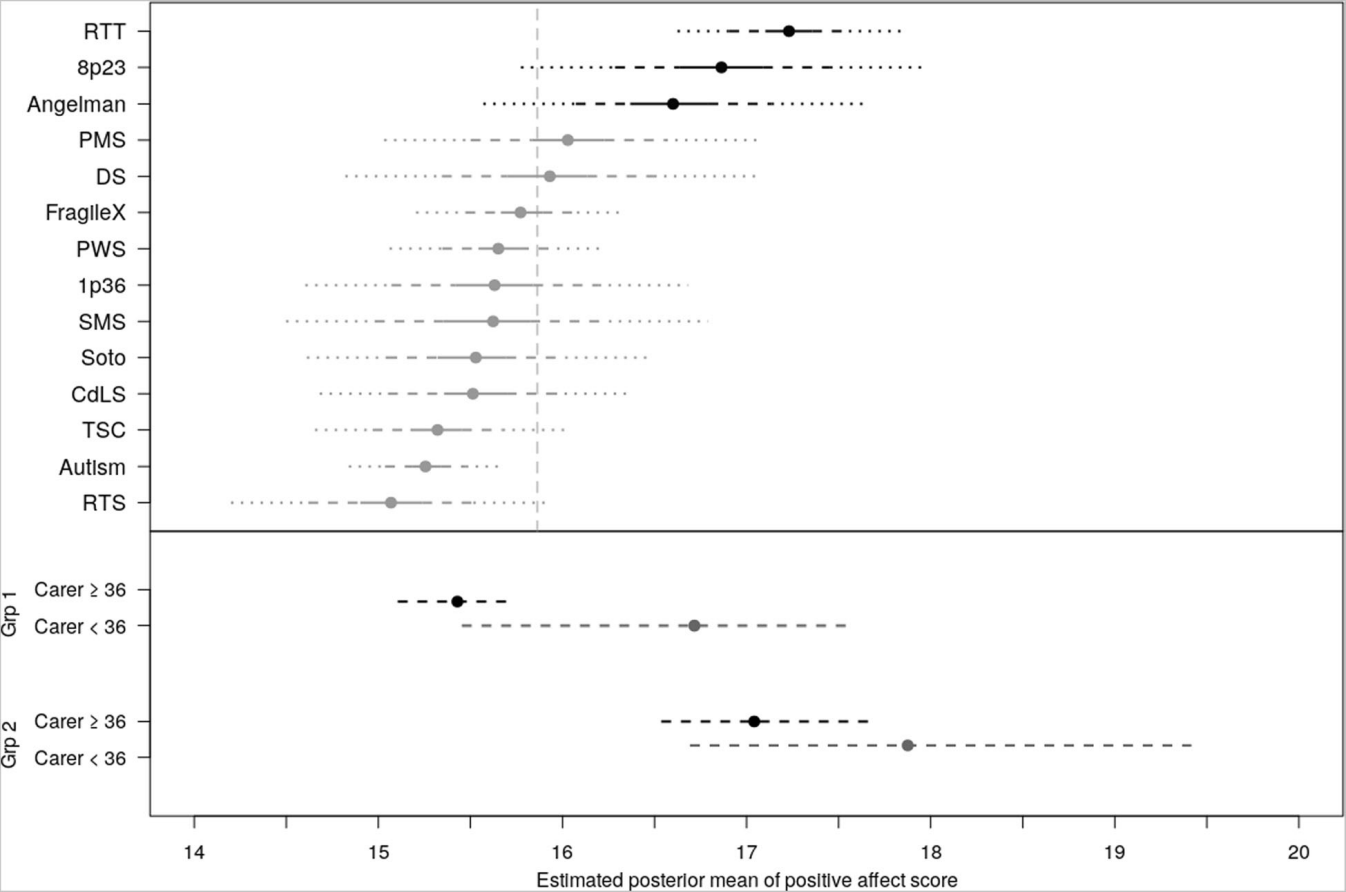
Positive Affect by syndrome group (upper panel) and by BMA groupings (lower panel). Upper panel: Estimated posterior mean scores for Positive Affect by syndrome group. Extended lines represent Credible Intervals: solid 20%, dashed 50%, dotted 80%. Vertical dashed line is mean of all participants. Lower panel: Posterior probabilities of Positive Affect based on BMA. Group 1: ASD, CdLS, DS, FXS, PMS, PWS, RTS, SMS, Soto, 1p36, TSC. Group 2: AS, RTT, 8p23

**Fig. 3:**
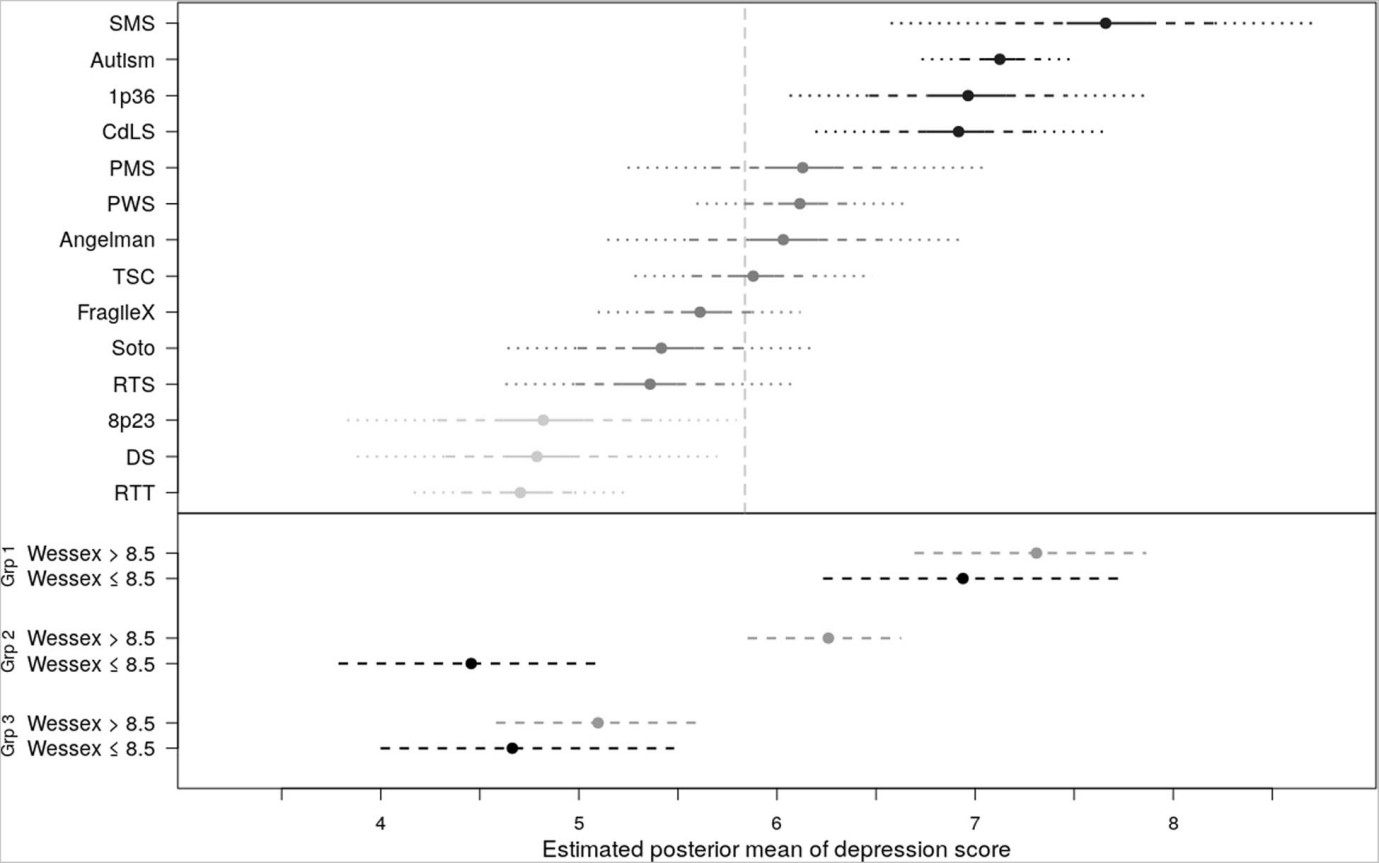
Depression by syndrome group (upper panel) and by BMA groupings (lower panel). Upper panel: Estimated posterior mean scores for Depression by syndrome group. Extended lines represent Credible Intervals: solid 20%, dashed 50%, dotted 80%. Vertical dashed line is mean of all participants.Lower panel: Posterior probabilities of Positive Gain based on BMA. Group 1: ASD, CdLS, SMS, 1p36, Group 2: AS, FXS, PWS, TSC, PMS, Group 3: DS, Soto, RTS, RTT, 8p23

**Fig. 4:**
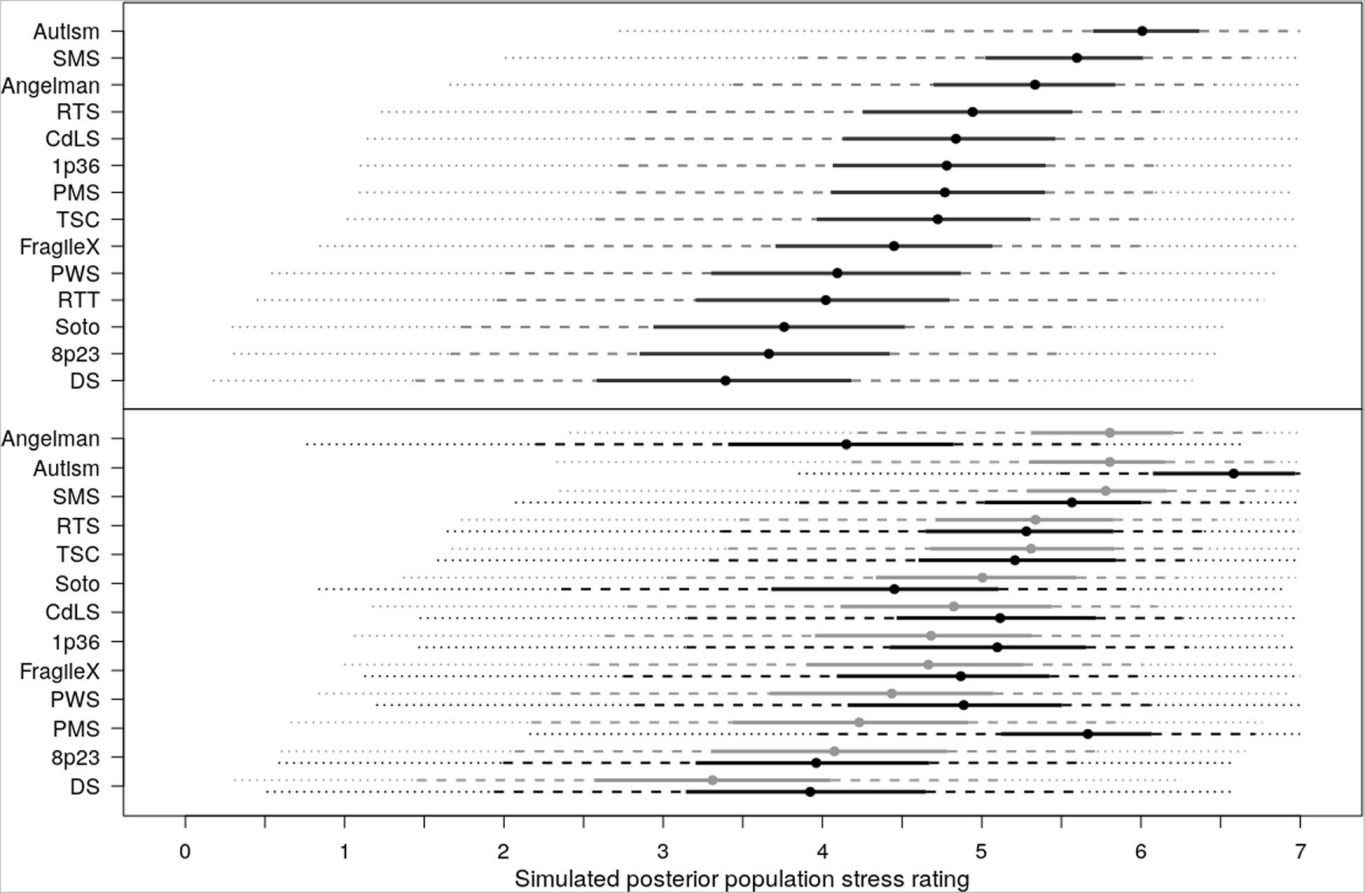
Stress by syndrome group (upper panel) and by posterior ratings (lower panel). Upper panel: Simulated posterior population scores for Stress Ratings by syndrome group. Extended lines represent Credible Intervals: solid 20%, dashed 50%, dotted 80%. Vertical dashed line is mean of all participants. Lower panel: Simulated posterior population stress rating of Positive Gain for children with Wessex self-help score below the maximum, split by child age. Grey line aged 8, black line age 16. Extended lines represent percentage of expected population: solid 20%, dashed 50%, dotted 80%

The simulated posterior distributions with credible intervals for each of the four measures are displayed in the upper panels of Figs. 1, 2, 3, 4. Data are presented in ascending order across the groups, meaning that the order of syndrome groups differs between each figure. Higher scores represent higher levels of the area being assessed, that is, higher levels of positive affect and positive gain, but also higher levels of depression and stress. Groupings (represented by shadings) are based on the posterior probabilities documented in Table 3. The CART diagrams are provided in the Additional file 1, items 1 to 4.

**Table 3:** Posterior estimates of mean (+ 90% credible interval) for continuous measure (median for stress) of maternal mental health with posterior probability of differing from autism

Syndrome	Positive Gain		Positive Affect		Depression			Stress	
	Mean (90% CrI)	Posterior prob. of difference with ASD	Mean (90% CrI)	Inclusion prob.	Mean (90% CrI)	Posterior prob. of difference with ASD.	Posterior prob. of being clinically depressed	Median (90% CrI)	Posterior prob. of difference with ASD
ASD	21.66 (20.99–22.33)	–	15.25 (14.83–15.67)	–	7.12 (6.63–7.62)	–	.51	6 (6–6)	–
AS	21.55 (20.20–22.89)	.56	16.60 (16.37–17.65)	.94	6.03 (4.88–7.18)	.93	.41	5 (5–6)	.95
CdLS	21.66 (20.57–22.81)	.49	15.51 (15.36–16.35)	.64	6.92 (6.00–7.90)	.67	.49	5 (4–6)	1
DS	22.42 (20.91–23.86)	.80	15.93 (15.70–17.07)	.76	4.79 (3.65–5.88)	1.00	.29	3 (2–5)	1
FXS	21.66 (20.27–22.42)	.51	15.77 (15.67–16.34)	.83	5.61 (4.94–6.26)	1.00	.37	4 (4–5)	1
PMS	22.98 (21.65–24.34)	.94	16.03 (15.83–17.06)	.83	6.13 (4.99–7.30)	.90	.41	5 (4–6)	1
PWS	21.06 (20.27–21.84)	.84	15.65 (15.54–16.23)	.77	6.12 (5.47–6.79)	.97	.41	4 (4–5)	1
RTT	22.71 (21.90–23.54)	.95	17.23 (17.10–17.86)	1	4.70 (4.01–5.38)	1.00	.29	4 (3–5)	1
RTS	22.70 (21.58–23.83)	.91	15.07 (14.90–15.91)	.60	5.36 (4.43–6.29)	1.00	.34	5 (4–6)	1
SMS	21.70 (20.13–23.33)	.52	15.62 (15.35–16.79)	.64	7.66 (6.28–9.02)	.74	.56	6 (5–6)	.83
Soto	21.78 (20.51–22.96)	.57	15.53 (15.32–16.49)	.63	5.42 (4.39–6.43)	.99	.35	4 (3–5)	1
TSC	21.64 (20.76–22.53)	.50	15.32 (15.17–16.01)	.54	5.88 (5.14–6.63)	.99	.39	5 (4–5)	1
1p36	21.64(20.28–23.05)	.51	15.63 (15.42–16.68)	.66	6.96 (5.84–8.15)	.57	.50	5 (4–6)	.99
8p23	21.89 (20.35–23.38)	.60	16.86 (16.64–17.96)	.96	4.82 (3.54–6.13)	1.00	.30	4 (2–5)	1

Posterior probability of difference = 1 indicates posterior distributions of estimate highly unlikely to be equivalent. Posterior probability = 0 indicates no detectable difference

### Positive mental health

#### Positive gain

The upper panel of Fig. 1 documents the simulated posterior distributions for positive gain with credible intervals across all syndrome groups and ASD. The CART informed the inclusion of syndrome group, child age (≤/> 8) and maternal age (≤/> 53) in the BMA. The results of the BMA (summarised graphically in the lower panel of Fig. 1) indicate two groups based upon syndrome (inclusion probability .75), with Group 1 (containing ASD, AS, CdLS, FXS, PWS, SMS, Soto, 1p36, 8p23 and TSC) reporting significantly lower positive gain than Group 2 (containing DS, RTS, PMS, RTT). Increasing child age (inclusion probability .41) and carer age (inclusion probability .36) had a mild negative influence on positive gain, with a slightly stronger impact of carer age in syndrome Group 2 than in Group 1 (inclusion probability .41).

#### Positive affect

The upper panel of Fig. 2 documents the simulated posterior distributions for positive affect with credible intervals across all syndrome groups and ASD. The CART informed the inclusion of syndrome group and maternal age (</ ≥36) into the BMA. The results of the BMA (summarised graphically in the lower panel of Fig. 2) indicate two groups based upon syndrome (inclusion probability .70), with Group 1 (containing ASD, CdLS, DS, FXS, PMS, PWS, RTS, SMS, Soto, 1p36, TSC) reporting significantly lower positive affect than Group 2 (containing AS, RTT, 8p23). Increasing carer age (inclusion probability .89) had a strong negative influence on positive affect, with a slightly stronger impact of carer age in syndrome Group 1 than in Group 2 (inclusion probability .37).

### Mental health difficulties

#### Depression

The upper panel of Fig. 3 documents the simulated posterior distributions for depression with credible intervals across all syndrome groups and ASD. The CART informed the inclusion of syndrome group and Wessex Score (=/< 9) into the BMA. The results of the BMA (summarised graphically in the lower panel of Fig. 3) indicate three groups based upon syndrome (inclusion probability .84 for Group 2, 1.0 for Group 3), with Group 1 (containing ASD, CdLS, SMS, 1p36) reporting significantly higher depression than Group 2 (containing AS, FXS, PWS, TSC, PMS), both of which report higher depression than Group 3 (containing DS, Soto, RTS, RTT, 8p23). Whether the child’s Wessex self-help score was at or below the maximum did not influence depression scores in Group 1 or Group 3, but had a significant influence on Group 2 (inclusion probability .77), with higher levels of depression in mothers of children who had a Wessex score below the maximum score of 9. It is important to note that depression scores in Group 1 are much higher than those for Groups 2 and 3, and as such, the Wessex score has limited ability to increase the depression rating.

As the HADS has a cut-off score for clinical levels of depression, the inclusion probability for mothers in each syndrome group being depressed was calculated and is presented in Table 3 (without consideration of child or carer variables). The syndromes in Group 1 of the BMA (ASD, CdLS, SMS, 1p36) have a 49–56% risk of scoring above the clinical cut-off, those in Group 2 (AS, FXS, PWS, TSC, PMS) a 37–41% risk, and those in Group 3 (DS, Soto, RTS, RTT, 8p23) a 29–35% risk.

#### Maternal stress

The upper panel of Fig. 4 documents the simulated posterior distributions for maternal stress with credible intervals across all syndrome groups and ASD. The CART identified syndrome group first, then Wessex (=/< 9) and finally child age (≤/> 8) as factors influencing maternal stress scores. The lower panel of Fig. 4 depicts simulated posterior mean scores for each syndrome only for those scoring below the maximum score of 9 on the Wessex self-help score. The data are divided by age group, with the grey line representing maternal stress levels for each syndrome when the child is age 8, and the black line representing each syndrome when the child is age 16. These variables were entered into the ordinal probit regression. Maternal stress tended to increase with child age (Bayesian posterior *p*-value = .01) with the exception of Soto (Bayesian posterior p-value <.01), AS (posterior p-value = .02), and SMS (Bayesian posterior p-value = .004) where it decreased with child age.

## Discussion

This is the first study exploring mental health and well-being in mothers of children with 13 rare genetic syndromes associated with neurodevelopmental disorders or disabilities in relation to each other and to mothers of children with ASD. It also provides the first published description of which we are aware of stress, depression and positive mental health of mothers of children with four syndromes: RTS, Soto, 1p36 and 8p23. The use of the Bayesian approach is both novel and important, as it allows for exploration of relative influences on different child or mother factors in relation to a range of maternal mental health measures whilst modelling for the uncertainty in this underresearched area.

The first key finding is that for different aspects of mental health, including positive mental health, different child and mother factors influenced the groupings of scores, and that the importance of these factors on maternal mental health is different for different syndromes. For example, the BMA for depression notes that mothers of children with some syndromes (ASD, CdLS, SMS, 1p36) had elevated levels of depression regardless of child ability or age, or age of the mother, and others had relatively low levels of depression (DS, Soto, RTS, RTT, 8p23) regardless of these variables. However, levels of maternal depression in AS, FXS, PWS, TSC and PMS showed a syndrome by ability interaction: mothers of children with the highest ability score on the Wessex had lower depression scores than mothers of children who had a score below the maximum on the Wessex (posterior mean depression ratings 4.46 and 6.26 respectively).

For maternal stress, the relationship was more complex, with the CART decision tree and regression both highlighting an interaction between Wessex score and syndrome, but also identifying the importance of child age within those with lower than maximum on the Wessex within a specific group of syndromes. For that group, stress tended to increase with child age: however, in AS, Soto and SMS, the opposite pattern was noted. Within positive mental health, carer and child age are important considerations, although the BMA again highlighted that these factors have different influences on different groups of syndromes for different aspects of positive mental health.

The second key finding is the identification of some syndromes where a notable proportion of mothers will be experiencing comparable levels of depression (SMS, 1p36, CdLS) and stress (SMS, AS) to mothers of children with ASD, a group that has always been highlighted within the literature as being at elevated risk for mental health difficulties. The prevalence rates of ASD in these syndromes vary (AS 34%, CdLS 43%, SMS 54% meet cutoff on the SCQ, 1p36 “few cases” [54–56]), and are comparable with some of the syndromes explored within this paper where mothers do not report comparable levels of mental health difficulties, for example, PMS 52.5–55%, Soto 83.3%, and RTT 61% [56–58]. Therefore, the similarities in mental health difficulties between these syndromes and ASD cannot be explained simply by the presence of “autism-symptoms” within these syndromes. This may suggest that different “direct effects” of a syndrome may have differing “indirect effects” in the context of the broader physical and cognitive phenotype. The finding that parents of different syndromes have elevated scores in different aspects of mental health difficulties is also of significance.

It is important to consider why maternal psychological well-being may be associated with child disability aetiology and why specific aetiologies interact differently with child and parent characteristics for certain aspects of maternal mental health. The research pertaining to parental stress in idiopathic intellectual disability and ASD has highlighted the impact of behavioural characteristics and behaviours that challenge on parental stress in both cross-sectional and longitudinal studies [59]. The presence of behaviours that challenge may be a significant factor contributing to the results reported in this study. However, the impact of behaviours that challenge on parent stress and well-being, over and above the syndrome, differs between different syndromes (e.g. [12]). It cannot therefore be assumed that the between-syndrome similarities and differences in parental mental health and well-being identified within this study can be solely explained by behavioural difficulties as “behaviour is but one aspect of aetiology-related characteristics that affect these children’s everyday lives” [60].

In their review of the literature, [60] highlight the importance of considering the “non-behavioural” aspects that may impact upon parental well-being including the child’s health status, the level of caregiving required, both parent and child personality, child facial characteristics and physical phenotype, and the timing and predictability-expectedness of problems. Parents of children with different syndromes have different concerns about their children [61] and there may be different levels of parental acceptance and understanding of the genetic cause (including aspects relating to hereditability) and societal acceptance of the genetic syndrome [11]. In qualitative interviews, mothers of young people with genetic syndromes reported that negative public reactions, difficulties with social inclusion, problems accessing social and medical services and a lack of accessible knowledge about the syndrome, were factors that increased maternal stress [62]. It is therefore critical that future explorations of maternal well-being in mothers of children with rare genetic syndromes consider the behaviour (or behavioural phenotype) within the broader subset of phenotypes of the syndrome, including the physical (including physical presentation and physical health) and cognitive phenotypes (including autism symptomatology). Now that the current study has documented that child and mother variables influence the profile of maternal mental health across a range of genetic syndromes, researchers can begin to model and explore further sources of variance, with a focus on child behaviour in the context of other child, parent and family characteristics. Just as [63, 64] developed syndrome-specific models of syndromes to behaviour, these results of further explorations could model the “indirect” effect of syndromes [19] to consider individual and systemic factors within individual or groups of syndromes. Although this is highlighted as an important avenue for future research, it is likely that such relationships will be complex and there will inevitably be variations both within and between syndromes.

Parents, syndrome support groups and professionals encourage the use of knowledge relating to genetic syndromes to tailor services and provide proactive services within syndrome-specific at-risk areas [32, 65]. The results of this study highlight potential factors in being able to begin to identify mothers of children with specific syndromes who may be particularly vulnerable to mental health problems, and raise the possibility of targeted and early support for families. Given that parental stress is associated with child progress and response to intervention [66], it is important for both clinicians and researchers within this field to be aware of parents who may be at increased risk of stress or mental health problems, and who as a result, may find it difficult to benefit from standard parenting interventions. As noted [67], the best approach may be to integrate stress or depression reduction techniques into the early stages of parent training packages for high-risk parents to maximise the effectiveness of the standard training intervention. Studies such as this may also assist with directing parents to syndrome-informed early support and helpful resources to develop effective support networks.

Our identification of specific factors that influence positive maternal health is novel. Positive mental health is not the opposite of mental health difficulties, and this is highlighted in the patterns within syndromes. For example, although the AS mothers are amongst the most stressed of the syndromes assessed, they are reportedly relatively high positive affect. Interestingly, unlike the data for stress and depression, level of ability (as measured by the Wessex) did not influence positive mental health but instead, syndrome, child and parent age were important. Although research on parental positivity in the field of IDD is still in its infancy, the present research suggests that there may be some within (genetic) disability group variation that should be further explored in relation to parent and child demographic variables.

There are several limitations to the present study. Firstly, there is a possible ascertainment bias, as parents were recruited through syndrome support groups. The method of recruitment did not allow for any details on non-responders, who may have potentially been experiencing more difficulties which prevented them from completing the questionnaire. There is also no assessment of socioeconomic status. However, these potential biases are likely to have applied across the genetic syndrome groups, and may have had limited impact on the cross-sectional comparison. Another limitation is that confirmation of genetic testing was not checked for each participant. Detailed genetic information on each participant would allow for further exploration of maternal mental health and well-being in relation to genetic subtypes and other factors such as hereditability. Whilst all parents were members of specific support groups, it was only possible to provide some level of validation within the ASD group (by only including participants who score above the cut-off on the SCQ, albeit not a diagnostic criterion).

Secondly, the study is cross-sectional and has a large sample with a large age range and so does not allow for comment on the course or progression of the maternal outcomes explored. The range of factors explored was also limited and as discussed above, further studies are needed to explore the relative impact of both behavioural and non-behavioural factors on parental well-being. Longitudinal studies of parents of children with idiopathic intellectual disability and those with autism suggest that parental stress may reduce as children age, but this is largely dependent upon help received and the presence or severity of challenging behaviour [68]. Longitudinal methodologies would allow exploration over time of the stability and course of the mental health of mothers of children with rare genetic syndromes and neurodevelopmental disorders [69] and are increasingly important given the suggestion of change in some behavioural phenotypes with age (e.g. [70]). Whilst it is arguably the only way in which to collect a sample of this size and breadth, the limitations of collecting information via informant questionnaires should be recognised, especially in relation to providing in-depth sample characterisation.

## Conclusions

In conclusion, this study uses Bayesian statistical modelling in a novel manner to highlight the level of variability in mental health amongst mothers of children with rare genetic disorders and neurodevelopmental disorders. To date, mothers of children with autism have been shown to be a high-risk group, experiencing the highest levels of stress and mental health problems of parents with children with neurodevelopmental disorders [71]. Such a perspective should now be broadened, as the data here show that there are notable proportions of mothers of children with specific genetic syndromes who experience levels of depression and stress similar to the levels reported by mothers of children with autism. Further research is needed to explore the broader parent and child characteristics, including behavioural and family characteristics that are contributing to such elevated mental health difficulties.

## Key points

- Little is known about the mental health of mothers of children with rare genetic syndromes
- Different aspects of maternal mental health are influenced by different child and mother factors
- Depression levels are comparable between mothers of children with Smith Magenis, 1p36 and Cornelia de Lange syndromes and autism
- Stress levels are comparable between mothers of children with Smith Magenis, Angelman syndromes and autism
- Further work is needed to explore the relationship between maternal mental health, syndromes and other child and mother variables.

## Additional file

Additional file 1: Classification and regression tree (CART) diagrams for each measure. (PDF 58 kb)

## Abbreviations

1p36: 1p36 deletion syndrome
8p23: 8p23 deletion syndrome
AS: Angelman syndrome
ASD: Autism Spectrum Disorder
CdLS: Cornelia de Lange syndrome
DS: Down syndrome
FXS: Fragile-X syndrome
PMS: Phelan McDermid syndrome
PWS: Prader-Willi syndrome
RTS: Rubenstein Taybi syndrome
RTT: Rett syndrome
SMS: Smith Magenis syndrome
TSC: Tuberous Sclerosis Complex
