# Supplementary material for: Using Bayesian methodology to explore the profile of mental health and well-being in 646 mothers of children with 13 rare genetic syndromes in relation to mothers of children with autism
Source: Orphanet J Rare Dis. 2018 Oct 25;13:185. doi: 10.1186/s13023-018-0924-1 (PMC6203267; doi:10.1186/s13023-018-0924-1)

Depression: Group 1 – highest risk of depression

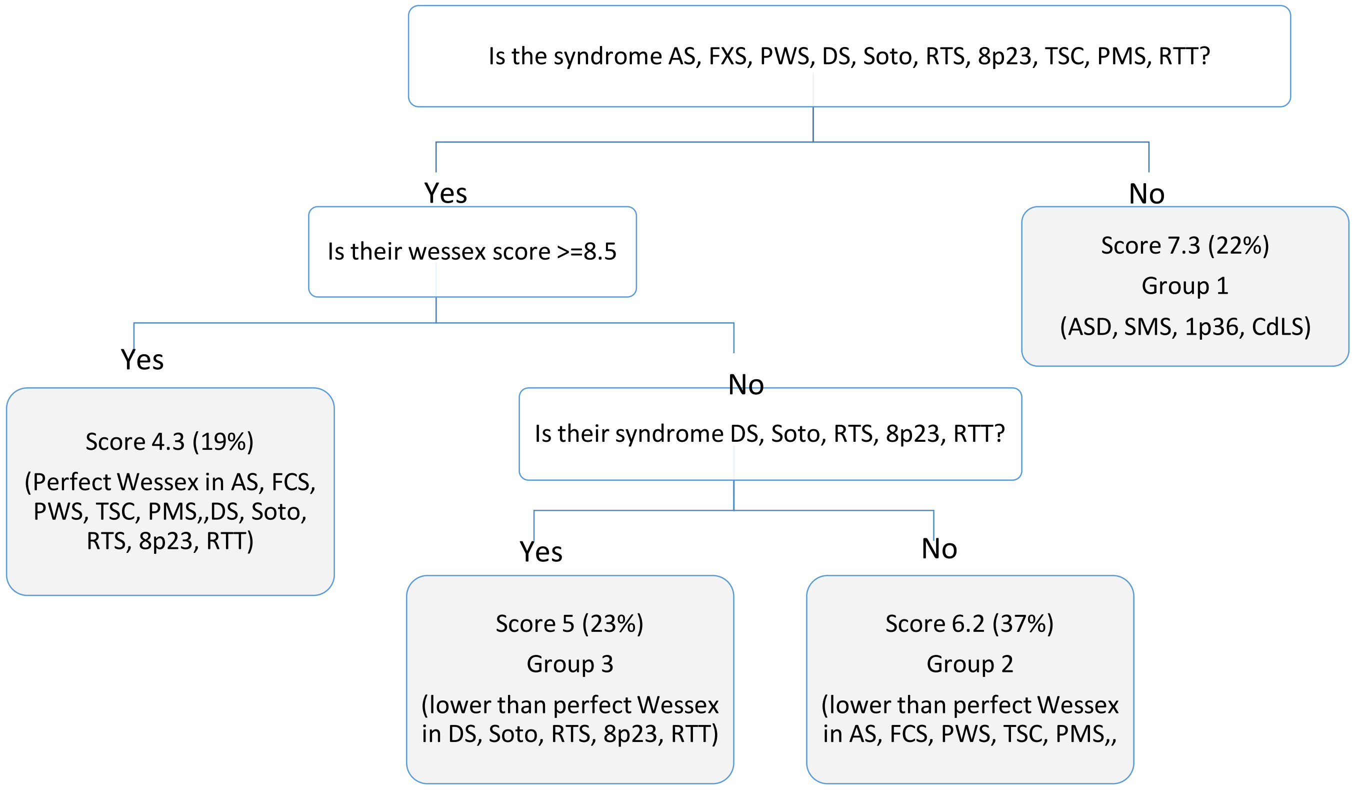

Stress (higher score, more stress)

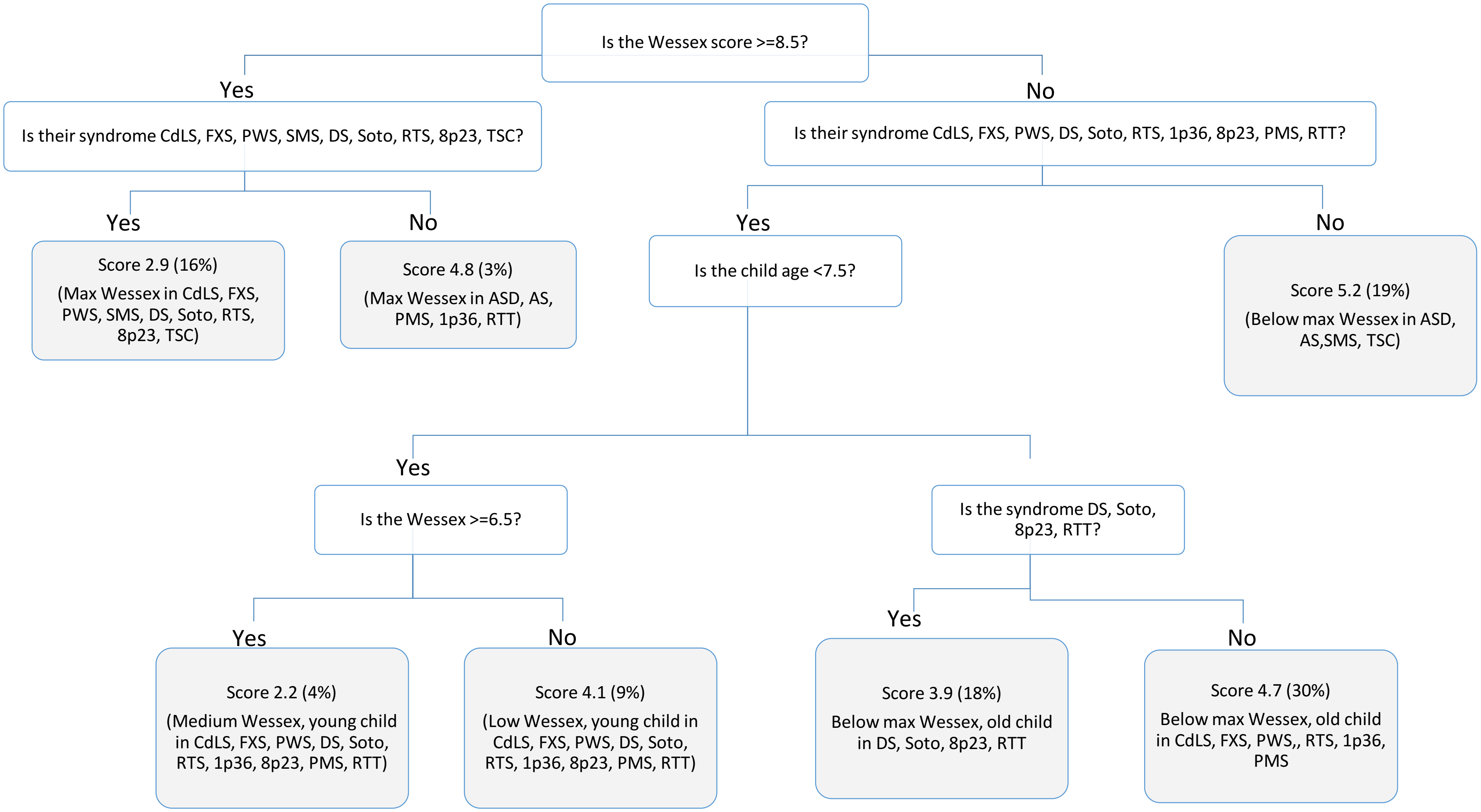

Positive Gain (higher score = more positive gain)

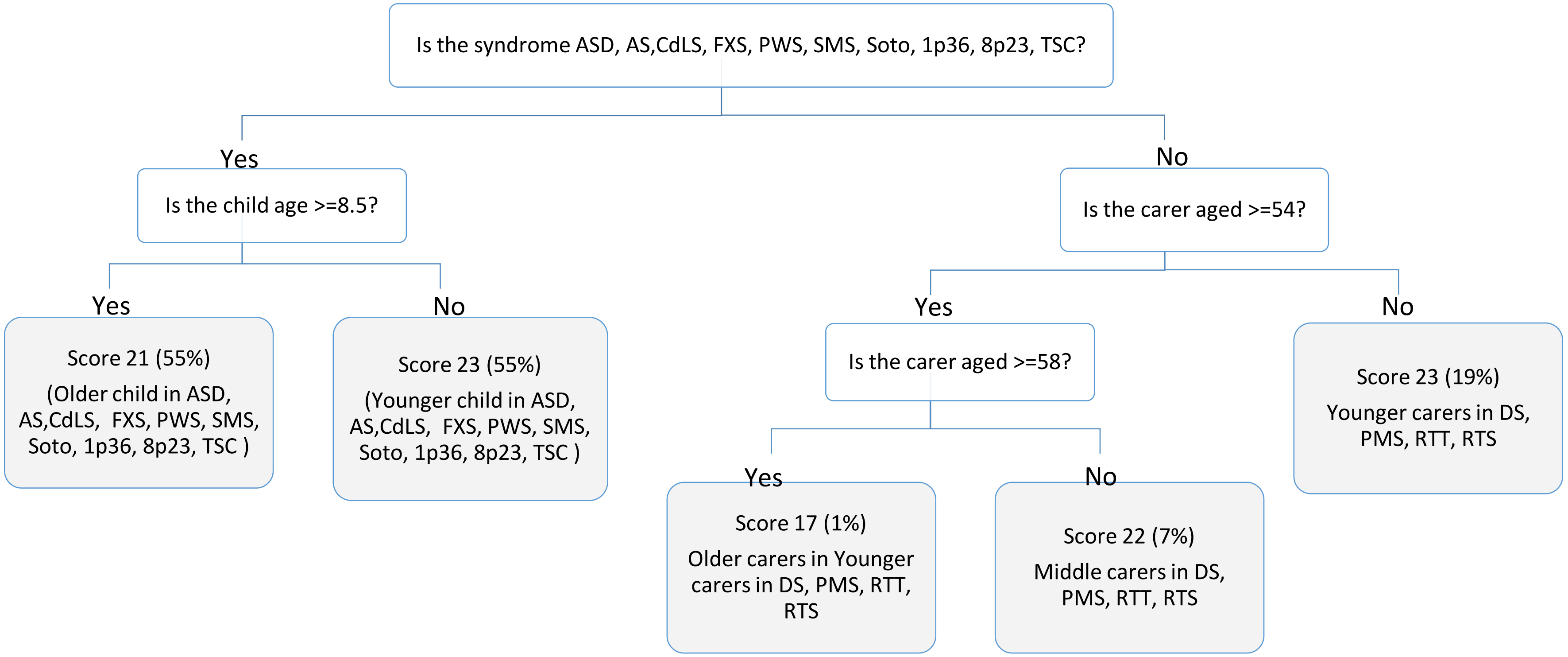

Positive affect – higher = more positive affect

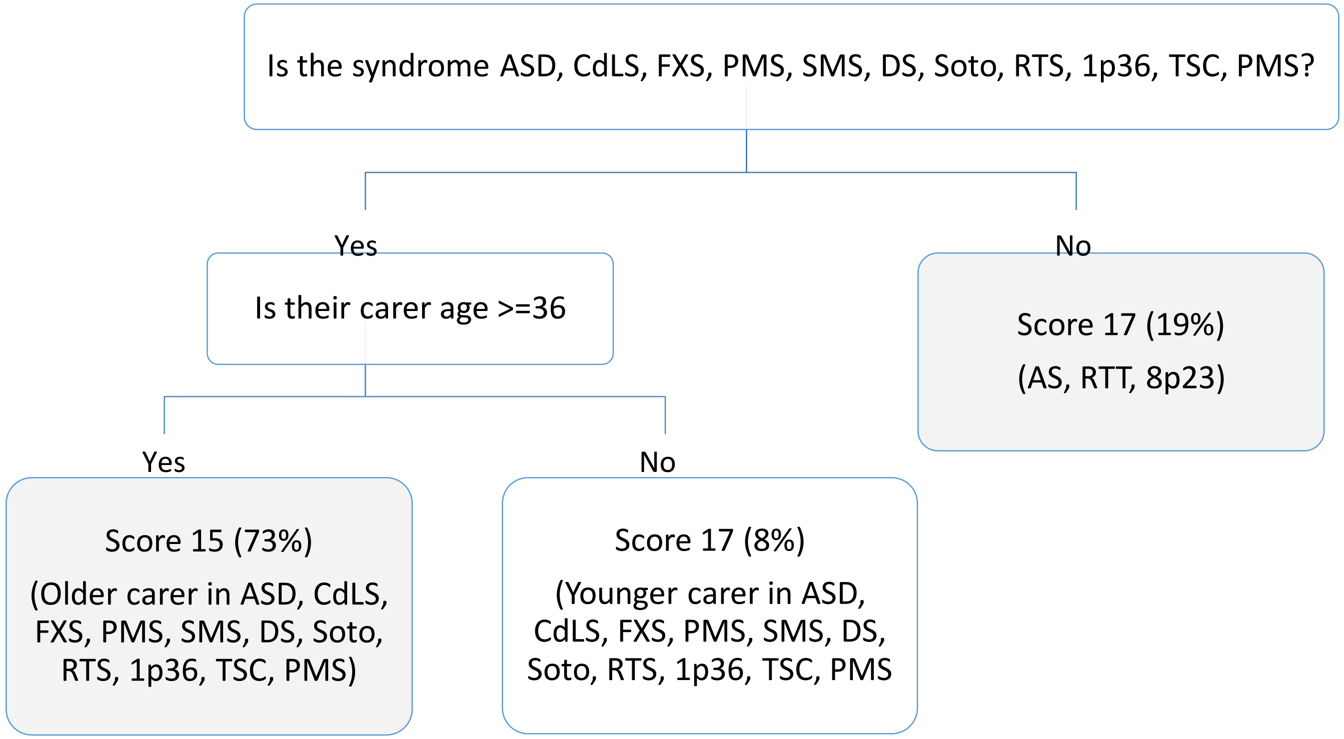

Supplement: Supplementary file 1 — Classification and regression tree (CART) diagrams for each measure. (PDF 58 kb) [file 13023_2018_924_MOESM1_ESM.pdf]
